# Antiplatelet and anticoagulant therapy in patients with submacular hemorrhage caused by neovascular age-related macular degeneration

**DOI:** 10.1007/s00417-022-05885-2

**Published:** 2022-11-29

**Authors:** Constance Weber, Maria Bertelsmann, Zoe Kiy, Isabel Stasik, Frank G. Holz, Raffael Liegl

**Affiliations:** 1 Department of Ophthalmology, University of Bonn, Ernst-Abbe-Str. 2, 53127 Bonn, Germany; 2 Department of Anesthesiology and Critical Care Medicine, University of Münster, Münster, Germany

**Keywords:** AMD, Antiplatelet therapy, Anticoagulant therapy, Blood thinners, Neovascular AMD, Submacular hemorrhage

## Abstract

**Purpose:**

Patients with extensive submacular hemorrhage (SMH) caused by age-related macular degeneration (AMD) have a poor visual prognosis despite surgical intervention. Systemic blood-thinning drugs, which are commonly prescribed in the same age group, are known to increase the risk of severe hemorrhage in many parts of the body. This study aimed to investigate whether systemic blood-thinning drugs have an impact on the severity of SMH and if there are differences between the different types of blood-thinning medication.

**Methods:**

We reviewed the medical records of patients who suffered from surgically treated SMH between 2020 and 2022. All patients received a full ophthalmologic examination upon presentation including best-corrected visual acuity (BCVA) and optical coherence tomography. Other characteristics that were recorded included size of hemorrhage, blood-thinning therapy, and reason for intake.

**Results:**

A total of 115 patients with a mean age of 82 years were included in this retrospective analysis. Eighty-three patients (72.2%) were on blood-thinning therapy. The mean size of SMH was 32.01 mm^2^. Mean BCVA at initial presentation was 1.63 logMAR and 1.59 logMAR 1 year after surgery. The size of SMH was significantly larger in patients on blood-thinning medication (35.92 mm^2^ vs. 21.91 mm^2^) (*p* = 0.001) and their BCVA postoperatively was worse with 1.68 logMAR compared to 1.30 logMAR after 1 year (*p* = 0.503). Patients with vitamin K antagonists had larger SMH size and worse outcomes regarding BCVA compared to direct oral anticoagulants.

**Conclusion:**

Blood thinners in patients with AMD affect the severity of SMH. Consequently, the indication for their intake should be critically evaluated.


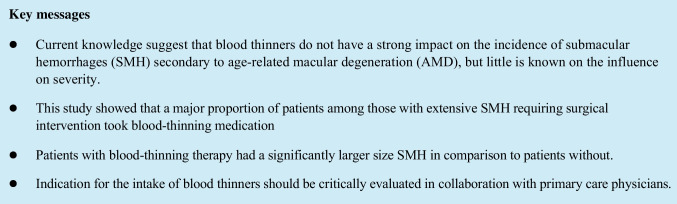


## Introduction

Age-related macular degeneration (AMD) is the most common cause for severe visual loss in industrialized countries. The incidence is expected to rise from approximately 67 million patients in 2020 to 77 million patients in 2050 [1].

One of the most devastating complications of neovascular AMD is the development of a submacular hemorrhage (SMH) secondary to macular neovascularization (MNV). The visual prognosis is typically guarded. Even after resolution of the hemorrhage, a remaining macular scar can cause permanent visual loss in a large proportion of patients [2]. Many of these elderly patients with AMD are concurrently treated with oral blood-thinning medication due to systemic comorbidities. There are two major types of blood-thinning treatments, i.e., antiplatelet and anticoagulant therapy.

Antiplatelet agents inhibit platelet activation and aggregation via the inhibition of receptors or enzymes, such as cyclooxygenases (COX) [3]. These represent an established therapeutic option for the prevention of cardiovascular events and are believed to reduce the mortality and morbidity rate of these patients. The most frequently used antiplatelet drug is acetylsalicylic acid (ASA) which is also considered to be the most frequently prescribed drug in the world [4–6]. ASA is recommended for secondary prevention of myocardial infarction, stroke, or transient ischemic attack [7, 8]. Even though there are no clear recommendations for the use of ASA as primary prophylaxis of cardiovascular events, it is prescribed very frequently for that cause [9, 10]. In patients with acute coronary syndrome after coronary stent implantation, a dual antiplatelet therapy (DAPT) represents the treatment of choice, combining ASA with an adenosine diphosphate receptor antagonist [11].

A study by O’Brien and colleagues reported that in the USA 23.4% of adults aged 40 years or over and nearly 50% of adults aged over 70 years took aspirin on a daily base. However, 22.8% of these adults did not have a physician’s recommendation for ASA [12].

Despite its advantages in the prevention and therapy of cardiovascular diseases, a therapy with antiplatelet agents poses an increased risk of bleeding [13]. There has been an ongoing debate on the risks and benefits of blood thinners. Recent studies are rather critical with regard to the increasing use of antiplatelet therapy, especially low-dose ASA. Even low-dose aspirin resulted in a significantly higher risk of major hemorrhages in several studies, including hemorrhagic stroke and gastrointestinal hemorrhage, without significantly reducing the risk of cardiovascular diseases in comparison to placebo [7, 14, 15].

Anticoagulant therapy is prescribed for patients with atrial fibrillation to reduce the risk of stroke or other thromboembolic events. The two most important anticoagulants are vitamin K antagonists (VKA) and direct oral anticoagulants (DOACs). Coumarins are vitamin K antagonists inhibiting the formation of vitamin K–related clotting factors. DOAC inhibit factor II or factor Xa of the coagulation cascade [16, 17]. Studies demonstrated that treatment with DOAC results in a decreased bleeding risk in comparison to VKA at a comparable efficacy [18, 19]. In Europe, patients with atrial fibrillation rather receive DOAC than VKA [20]. However, after heart valve replacement, VKA represents the first-line therapy as DOACs are not approved for this indication.

Many patients suffering from AMD are concomitantly on blood-thinning therapy. Studies investigating the role of blood thinners in AMD patients suggest that a blood-thinning therapy does not increase the incidence of subretinal hemorrhages [21]. However, only a few studies reported that blood-thinning medication has an influence on the extent of SMH, particularly in patients suffering from arterial hypertension [22, 23]. Studies investigating possible differences between the different types of blood-thinning medication are lacking.

This retrospective study examined whether patients who developed SMH requiring surgical intervention were more likely to be taking concomitant blood thinners. In addition, this study investigated whether the use of blood thinners had an impact on the extent of SMH and whether there were differences between antiplatelet agents and anticoagulants or between VKA and DOAC.

## Material and methods

### Patients

Medical records of all patients who presented with a submacular hemorrhage caused by neovascular AMD at the Department of Ophthalmology, University of Bonn, Germany, between January 2020 and March 2022 were retrospectively reviewed. Patients with submacular hemorrhages caused by other pathologies, e.g., retinal arterial macroaneurysms, were excluded. There were no limits regarding age, gender, or ethnicity. Treatment consisted either in intravitreal injection (IVI) of recombinant tissue plasminogen activator (rtPA), bevacizumab, and gas tamponade or pars plana vitrectomy (ppV) with subretinal rtPA administration and intravitreal bevacizumab injection with the choice of tamponade upon the surgeon’s discretion. All SMHs were caused by neovascular AMD. We included patients with a submacular hemorrhage which involved the foveal center amenable to surgical displacement and which was large enough to require surgical intervention, as it was defined before by Sharma et al. [24]. A submacular hemorrhage within the arcades not exceeding four disc areas was treated with intravitreal rtPA and bevacizumab injection. Larger SMHs received ppV. Symptoms of SMH due to AMD included acute visual deterioration, appearance on funduscopy, optical coherence tomography (OCT) imaging, and fundus photography.

All patients received a full ophthalmologic examination, including the assessment of the best-corrected visual acuity (BCVA) (measured in Snellen decimal visual acuity and converted to logMAR for statistical analyses), measurement of intraocular pressure, slit-lamp biomicroscopy, funduscopy, optical coherence tomography, and fundus photography. The recorded data included preoperative clinical features, details of the surgical procedure, systemic medical treatment, especially regarding blood-thinning medication and comorbidities, and follow-up regarding the BCVA.

Measurements of the size of SMH were performed using the display mode of the Spectralis OCT (Heidelberg Engineering, Germany). If the SMHs exceeded the area that the OCT was able to scan or if the quality was low, we measured the size on fundus photography. All analyses were conducted on a deidentified data set. A waiver of the local ethics committee was granted due to the retrospective character of this study. The study protocol conforms to the ethical guidelines of the 2000 Declaration of Helsinki as reflected in a priori approval by the institution’s human research committee.

### Statistical analyses

Statistical analyses were performed with SPSS Statistics version 27.0.0 (IBM Corporation, New York). The Pearson’s chi-square test was used to compare the distributions of the nominal- or ordinal-scaled variables. The *t*-test was used for normal distributions and Mann–Whitney *U*-test was used for non-normal distributions in order to compare independent groups. Correlation was tested using Spearman’s Rho. All tests were performed two-sided and we considered *p* values < 0.05 to be statistically significant.

## Results

Included in this study were a total of 115 patients who presented to the Department of Ophthalmology, University of Bonn, Germany, with a SMH due to AMD and who received either an intravitreal injection of recombinant tPA, bevacizumab, and gas tamponade or ppV with subretinal rTPA administration and intravitreal bevacizumab injection, followed by gas tamponade. The mean age was 82.2 years (range, 66.2–101.5 year). Seventy women (60.9%) and 45 men (39.1%) were included. The characteristics of our patient cohort are described in Table 1.

**Table 1:** Patient characteristics

	All patients	
	(*n* = 115)	%
Gender		
Female/male	70/45	60.9/39.1
Age		
Mean (range)	82.2 (66–101)	
Lens status		
Phakic/pseudophakic	26/81	24.3/75.7
IVI therapy before SMH		
Yes/no	65/50	57.4/42.6
Blood-thinning therapy		
Yes/no	83/32	72.2/27.8
If yes		
Antiplatelet therapy	46	40.0
- ASA	40	34.8
- Clopidogrel	4	3.5
- DAPT	2	1.7
Anticoagulation	37	32.2
- DOAC	32	27.8
- Vitamin K antagonist	4	3.5
- Heparin	1	0.9
Arterial hypertension		
Yes/no	72/42	63.2/36.8
Atrial fibrillation		
Yes/no	27/88	23.5/76.5
Status post cardiac stent-implantation		
Yes/no	8/107	6.8/93.2
Status post stroke		
Yes/no	3/112	2.6/97.4
Status post venous thrombosis or pulmonary embolism		
Yes/no	2/113	1.7/98.3
Size of submacular hemorrhage		
Mean (range)	32.01 (2.43–90.81)	
Surgical details		
rtPA subretinal via vitrectomy		
Yes/no	67/48	58.3/41.8
rtPA intravitreal		
Yes/no	48/67	41.8/58.3
Endotamponade		
Gas (SF6)	100	87.0
Silicone oil	5	4.3
Air	10	8.7
Combined cataract surgery		
Yes/no	23/92	20.0/80.0
BCVA baseline (logMAR)		
Mean (range)	1.63 (0.4–2.7)	
BCVA 6 weeks (logMAR)		
Mean (range)	1.54 (0.1–2.7)	
BCVA 6 months (logMAR)		
Mean (range)	1.67 (0.2–2.7)	
BCVA 1 year (logMAR)		
Mean (range)	1.59 (0.4–2.7)	
BCVA fellow eye baseline (logMAR)		
Mean (range)	0.69 (0–3.0)	

The mean review period was 7.63 months (range, 0–19 m). Follow-up regarding BCVA was completed for 110 patients after 6 weeks, 91 patients after 6 months and 83 patients after 1 year. Seventy-two patients (62.6%) suffered from arterial hypertension and 22 patients (19.1%) from atrial fibrillation. Eighty-three patients (72.2%) were on blood-thinning medication whereas 32 patients (27.8%) were not. Thirty-seven patients (32.2%) received anticoagulant therapy, of these 32 patients (27.8%) with DOAC, 4 patients (3.5%) with VKA, and 1 patient with heparin (0.9%). Causes included permanent atrial fibrillation (27 patients, 22.9%), status post stroke (3 patients, 2.5%), and venous thrombosis or pulmonary embolism (2 patients, 1.7%). Forty-six patients (40.0%) received antiplatelet therapy, of these 40 patients (34.8%) with ASA, 4 patients (3.5%) with clopidogrel and 2 patients (1.7%) with DAPT. Reasons for antiplatelet therapy were cardiac stent implantation (8 patients, 6.8%) and no known reasons (38 patients, 33.1%).

All patients suffered from nAMD and presented with a SMH as defined before. The mean size of the SMH was 32.01 mm^2^ (2.43–90.81 mm^2^). Sixty-five patients (61.9%) had received IVI before the development of the SMH. There was no significant correlation between size of SMH and age (correlation coefficient: 0.095; *p* = 0.313).

All patients in our cohort were treated with intravitreal or subretinal rtPA. Sixty-seven patients (58.3%) received a pars plana vitrectomy. One hundred patients (87.0%) received a gas tamponade, and 9 patients (7.8%) a tamponade with silicone oil which was removed after a mean interval of 3.4 months. The choice of tamponade was based upon every surgeon’s discretion. Silicone oil was instilled in cases of more extensive hemorrhages and cases where intraoperatively active bleeding was suspected or observed.

BCVA at initial presentation was 1.63 logMAR (0.4–2.7 logMAR). The BCVA of the fellow eye was 0.69 logMAR (0 logMAR–2.7 logMAR). During the postoperative course, the BCVA was 1.54 logMAR 6 weeks, 1.68 logMAR 6 months, and 1.59 12 months after surgery.

There was a moderate correlation between size of SMH and visual acuity (correlation coefficient: 0.385; *p* < 0.001).

There was no significant difference concerning visual acuity at baseline in patients that had received IVI therapy before (affected eye 1.64 logMAR, fellow eye: 0.75 logMAR) in comparison to patients without prior IVI therapy (affected eye 1.67 logMAR, fellow eye 0.62 logMAR) (*p* = 0.81 and *p* = 0.39 respectively).

In order to evaluate the influence of blood-thinning medication, we compared patients on blood thinners (group 1a, 83 pat., 72.2%) to those without blood-thinning medication (group 1b, 32 pat., 27.8%) (Table 2).

**Table 2:** Comparison of patients with and without antiplatelet or anticoagulant therapy (blood thinners)

	Patients with blood thinners: *n* = 83 (%)	Patients without blood thinners: *n* = 32 (%)	*p* value
Gender			0.133
Female/male	47/36 (56.6/43.4)	23/9 (71.9/28.1)	
Age			0.057
Mean (range)	82.88 (66.2–101.4)	80.31 (68.3–98.0)	
Arterial hypertension			0.007
Yes/no	58/24 (70.7/29.3)	14/18 (43.8/56.3)	
Atrial fibrillation			0.001
Yes/no	27/57 (32.4/68.6)	0/31 (0/100)	
Size of submacular hemorrhage (mm^2^)			0.001
Mean (range)	35.92 (3.09–90.81)	21.90 (2.43–67.14)	
BCVA baseline (logMAR)			0.110
Mean (range)	1.71 (0.5–2.7)	1.49 (0.4–2.3)	
BCVA 6 weeks (logMAR)			0.060
Mean (range)	1.66 (0.4–2.7)	1.24 (0.1–2.3)	
BCVA 6 months (logMAR)			0.637
Mean (range)	1.72 (0.8–2.7)	1.60 (0.2–2.3)	
BCVA 1 year (logMAR)			0.503
Mean (range)	1.67 (0.8–2.7)	1.30 (0.4–2.3)	

There were no significant differences between both groups regarding age (*p* = 0.061) or gender (*p* = 0.136).

Patients on blood thinners were significantly more often affected by arterial hypertension (*p* = 0.007) and atrial fibrillation (*p* = 0.001). The size of SMH (35.92 mm^2^) was significantly larger in patients with blood-thinning medication compared to patients without (21.91 mm^2^) (*p* = 0.001). With 1.71 logMAR, BCVA at initial presentation was worse in group 1a as compared to group 1b with 1.49 logMAR; however, this difference was not significant. There were no differences concerning the surgical intervention with a similar amount of vitrectomies (*p* = 0.745) and choice of tamponade (*p* = 0.719). BCVA during the postoperative course was worse in group 1a with 1.67 logMAR than in group 1b with 1.24 logMAR at 6 weeks (*p* = 0.060) and with 1.68 logMAR and 1.30 logMAR consecutively at 1 year after surgery (*p* = 0.503).

We performed a subgroup analysis between patients on antiplatelet therapy (group 2a, 46 pat., 40.0%) and on anticoagulant therapy (group 2b, 37 pat., 32.2%) (Table 3).

**Table 3:** Comparison of patients with antiplatelet and anticoagulant therapy

	Patients with antiplatelet therapy: *n* = 46 (%)	Patients with anticoagulant therapy: *n* = 37 (%)	*p* value
Gender			0.078
Female/male	17/20 (45.9/54.1)	30/16 (65.2/34.8)	
Age			0.089
Mean	83.07 ( 70.2–95.2)	82.7 (69.4–90.81)	
Arterial hypertension			0.821
Yes/no	25/11 (69.4/30.6)	33/13 (71.7/28.3)	
Atrial fibrillation			0.0001
Yes/no	0/44 (0/100)	27/10 (72.9/27.1)	
Size of submacular hemorrhage			0.604
Mean (range)	34.49 (3.09–90.81)	37.06 (4.21–90.11)	
BCVA baseline (logMAR)			0.241
Mean (range)	1.82 (1.0–2.7)	1.56 (1.0–2.7)	
BCVA 6 weeks (logMAR)			0.241
Mean (range)	1.82 (0.5–2.7)	1.55 (0.8–2.7)	
BCVA 6 months (logMAR)			0.099
Mean (range)	2.00 (0.6–2.7)	1.47 (0.8–2.3)	
BCVA 1 year (logMAR)			0.097
Mean (range)	2.05 (0.8–2.7)	1.28 (0.8–2.7)	

There were no significant differences regarding age (*p* = 0.809), gender (*p* = 0.078), or the incidence of arterial hypertension (*p* = 0.821) between both groups. The size of SMH was similar with 37.06 mm^2^ in group 2a and 34.49 mm^2^ in group 2b (*p* = 0.604). There were no significant differences regarding BCVA at initial presentation (*p* = 0.143) or during the postoperative course at 6 weeks (*p* = 0.241) or 1 year (*p* = 0.097) after surgery.

In order to evaluate possible differences between the two major groups of anticoagulants, we conducted another subgroup analysis between patients on DOAC (group 3a, 32 pat., 28.7%) or VKA (group 3b, 4 pat., 3.5%) (Table 4).

**Table 4:** Comparison of patients with direct oral anticoagulants and vitamin K antagonists

	Patients with DOAC: *n* = 32 (%)	Patients with VKA: *n* = 4 (%)	*p* value
Gender			0.345
Female/male	16/16 (50/50)	3/1 (75/25)	
Age			0.770
Mean	82.77 (79.3–86.5)	83.513 (72.5–95.2)	
Arterial hypertension			0.849
Yes/no	23/9 (71.9/28.1)	2/2 (50/50)	
Atrial fibrillation			0.909
Yes/no	19/13 (59.3/40.7)	4/0 (100/0)	
Size of submacular hemorrhage			0.005
Mean (range)	31.75 (4.21–90.1)	63.70 (30.73–90.81)	
BCVA baseline (logMAR)			0.079
Mean (range)	1.77 (0.4–2.7)	2.4 (2.3–2.7)	
BCVA 6 weeks (logMAR)			0.411
Mean (range)	1.78 (1.0–2.7)	2.3 (2.3–2.3)	
BCVA 6 months (logMAR)			0.630
Mean (range)	1.95 (1.0–2.7)	2.3 (2.3–2.3)	
BCVA 1 year (logMAR)			0.264
Mean (range)	1.86 (1.0–2.7)	2.4 (2.3–2.7)	

There were no significant differences regarding age (*p* = 0.770), gender (*p* = 0.345), or the incidence of arterial hypertension (*p* = 0.849) between both groups. The size of SMH was significantly bigger in patients on VKA with 63.70 mm^2^ in comparison to patients on DOAC with 31.76 mm^2^ (*p* = 0.005). The BCVA was better in group 3a with 1.77 logMAR in comparison to group 3b with 2.40 logMAR at initial diagnosis (*p* = 0.079) and remained superior during the postoperative course with a BCVA of 1.79 logMAR in group 3a and of 2.30 logMAR in group 3b at 6 weeks after surgery (*p* = 0.411).

## Discussion

Ischemic heart disease, stroke, and cerebrovascular diseases are among the leading causes of death and are particularly common in people of older age [25]. All of these conditions usually require long-term use of blood-thinning medication. In the same group of patients, AMD and in particular its neovascular form is the leading cause of severe visual loss and the number of affected patients is expected to rise continuously over the next decades [1]. One of the most feared complications in patients with neovascular AMD is an extensive SMH [26].

All patients in this study had major SMH that needed surgical management and were treated by either IVI with rtPA and anti-VEGF with gas or a vitrectomy with subretinal injection of rtPA, IVI of an anti-VEGF drug and choice of tamponade upon the surgeon’s discretion.

In our study, mean visual acuity remained low even after surgical intervention with a BCVA of 1.63 logMAR preoperatively and 1.54 logMAR after 6 weeks and 1.59 logMAR after 1 year. Our data is in line with other studies supporting the assumption that after surgical treatment only a small improvement in visual acuity can be expected, particularly in patients with extensive SMH with low initial BCVA [2, 27, 28]. We showed that there was a moderate correlation between sizes of SMH and BCVA, which supports the assumption that more extensive SMHs come along with worse visual acuity outcome. Visual deterioration, especially bilaterally, has a significant impact on patients’ quality of life [29–32].

There was no significant difference concerning visual acuity at baseline in patients that had received IVI therapy before in comparison to patients without, though there was a trend for better visual acuity in patients without prior IVI therapy of the fellow eye. It is important to keep in mind that patients that had been treated for AMD might already have poorer visual acuity while patients without prior IVI would experience a more sudden vision loss. Due to the retrospective character of the study, we could not evaluate the visual acuity values before the SMH occurred in order to prove that assumption however.

In our cohort, almost 73% of all patients were on blood thinners and had a mean age of 82 years. Only few studies have been published regarding the intake of blood thinners and their impact on submacular hemorrhages in AMD patients. Mostly, it is stated that the development of SMH was not associated with the intake of blood-thinning medication; however, patients with arterial hypertension and concomitant blood-thinning therapy carried a higher risk [21, 23]. It seems reasonable that the use of a blood thinner per se is not necessarily associated with a more frequent occurrence of hemorrhages. In fact, the occurrence of small subretinal hemorrhages secondary to neovascular AMD is rather common and independent of systemic medication. The question arises whether patients receiving blood-thinning therapy are at a higher risk for extensive hemorrhages since small hemorrhages might be resorbed without a considerable effect on visual acuity. For that reason, only patients with SMH that were large enough to require surgical intervention were included in our study. The considerably greater proportion with almost 73% of patients with blood-thinning medication in our cohort exceeded the numbers reported in other neovascular AMD studies. Buijtendijk and colleagues investigated a cohort of 330 neovascular AMD patients and reported that only 40.9% of these were on blood-thinning medication [23]. In the post hoc analysis of the CATT trial, 52.4% of the 1165 patients were found to take blood-thinning medication [21]. Most of these studies reported no increased incidence of SMH in connection with blood-thinning medication. However, none of these studies specifically evaluated whether patients on blood thinners suffered from more extensive submacular hemorrhages. In our cohort, SMH areas were significantly larger in patients on blood thinners than in patients without.

However, the number of vitrectomies was not significantly different in both groups. This is due to the fact that we performed a vitrectomy in patients with SMH larger than four disc areas (approximately 10 mm^2^). The mean size of SMH in patients without blood thinners was 21.91 mm^2^ and 35.92 mm^2^ in patients with blood-thinning medication.

Our data suggests that patients concomitantly taking blood thinners have a higher risk of developing extensive SMH. Data availability on how many people are actually on blood-thinning medications in Germany and other regions of the world is scarce, but it is estimated that approximately one million people in Germany are taking blood thinners regularly, especially antiplatelets such as ASA. Further, VKA such as coumarins were among the most commonly prescribed drugs in patients with atrial fibrillation. However, they have been superseded by DOAC after their introduction [33].

In order to evaluate possible differences among blood-thinning medications, we performed two subgroup analyses. To the best of our knowledge, studies investigating possible differences between the different types of blood-thinning medication are lacking. The number of patients with antiplatelet (40.0%) and anticoagulant therapy (32.2%) were similar and both groups showed a comparable size of SMH. Even though indications for both medications vary, our findings suggest that the use of antiplatelets has a similar impact and risk with regard to the severity of SMH as a therapy with anticoagulants. This finding is of importance since most patients under anticoagulant therapy have fixed indications necessitating the therapy. There seems to be a lower threshold, however, when it comes to prescribing or recommending antiplatelets. In our cohort, 38 of 46 patients with antiplatelet therapy were not able to tell why they were taking antiplatelet drugs nor were they diagnosed with a cardiovascular comorbidity that clearly indicated this medication. A study by O’Brien et al. stated that nearly 50% of adults over the age of 70 years were taking aspirin daily. Of these, nearly 25% did not have a clear recommendation for this therapy [12]. ASA used to be viewed as a highly effective drug for the prevention of cardiovascular events with a manageable risk profile. Recently, many studies have been published raising doubts about the effectiveness of ASA and reporting on significantly more hemorrhagic events in patients on ASA. A prospective study by McNeil and colleagues included 19,114 patients who either received 100 mg of ASA or a placebo. The rate of cardiovascular events was similar in both groups while the group on ASA developed significantly more major hemorrhages [15].

Not every patient with atrial fibrillation requires anticoagulant therapy. Several tools exist to determine the need for anticoagulant therapy and balance the general risk of bleeding. The CHA2DS2-VASc score is a useful tool to assess whether a patient with atrial fibrillation actually needs anticoagulant therapy and to estimate the risk of developing a stroke. The HAS-BLED score additionally helps to assess the risk of bleeding. Having these tools in mind, we believe that the indication for anticoagulant therapy should be well-weighed and discontinuation of blood-thinning therapy should be discussed in certain cases. To take care of the fact that DOAC showed a generally decreased bleeding risk in comparison to VKA [18, 19], we performed a subgroup analysis for patients on DOAC and on VKA in order to account for possible differences in our cohort. Patients on DOAC had a significantly smaller size of SMH than patients on VKA and showed a better visual development during the postoperative course. However, these findings need to be interpreted with caution since the number of patients with VKA (*n* = 4) was too small to draw definite conclusions. In Europe, the ratio has already shifted in favor of more prescriptions of DOAC than VKA [20]. It will be of great interest to elaborate on this topic in further studies.

A close communication between general practitioners, internists, cardiologists, and ophthalmologists is desirable in order to treat patients holistically. Most physicians who are not ophthalmologists might not be aware of neovascular AMD as a comorbidity of their patients and thus may not consider the risk of an extensive submacular hemorrhage.

One of the limitations of this study is its retrospective nature. Further, when comparing DOAC and VKA, the number of people with VKA is rather low in order to account for statistical significant differences and robust conclusion. Moreover, we did not perform a regression analysis for other comorbidities, such as arterial hypertension, which might have an impact on the development and size of SMH. In order to address these limitations, it would be useful to perform prospective studies in the future.

It seems reasonable that the intake of blood thinners has no major impact on the incidence of subretinal hemorrhages caused by AMD since they occur sporadically. However, our data show that among patients with an extensive SMH that needed surgical treatment, nearly three quarter were under the intake of blood-thinning therapy. Patients with blood thinners developed significantly larger SMH. This leads to the assumption that antiplatelet and anticoagulant therapies in patients with AMD affect the severity of submacular hemorrhages. Consequently, the indication for their intake should be critically evaluated in these patients.

## Declarations

All analyses were conducted on a deidentified data set. A waiver of the local ethics committee was granted due to the retrospective character of this study. The study protocol conforms to the ethical guidelines of the 2000 Declaration of Helsinki as reflected in a priori approval by the institution’s human research committee.
